# Ferrostatin-1 facilitated neurological functional rehabilitation of spinal cord injury mice by inhibiting ferroptosis

**DOI:** 10.1186/s40001-023-01264-7

**Published:** 2023-09-11

**Authors:** Zhenhai Zhou, Hao Luo, Honggui Yu, Zhiming Liu, Junlong Zhong, Jiachao Xiong, Kai Cao

**Affiliations:** 1 The Orthopaedic Hospital, The First Affiliated Hospital of Nanchang University, #1519 Dongyue Avenue, Nanchang, 330052 China; 2 Institute of Spine and Spinal Cord, Nanchang University, Nanchang, China

**Keywords:** Ferrostatin-1, Spinal cord injury, Neurological functional rehabilitation, Ferroptosis, Nrf2/HO-1

## Abstract

**Background:**

To seek the potential therapy for spinal cord injury, Ferrostatin-1, the first ferroptosis inhibitor, was administrated in spinal cord injury mice to identify the therapeutic effect.

**Methods:**

Spinal cord injury model was established by a modified Allen’s method. Then, ferrostatin-1 was administrated by intraspinal injection. Cortical evoked motor potential and BMS were indicated to assess the neurological function rehabilitation. H&E, Nissl’s staining, NeuN, and GFAP immunofluorescence were used to identify the histological manifestation on the mice with the injured spinal cord. Spinosin, a selective small molecule activator of the Nrf2/HO-1 signaling pathway, was administrated to verify the underlying mechanism of ferrostatin-1.

**Results:**

Ferrostatin-1 promoted the rehabilitation of cortical evoked motor potential and BMS scores, synchronized with improvement in the histological manifestation of neuron survival and scar formation. Spinosin disturbed the benefits of ferrostatin-1 administration on histological and neurobehavioral manifestation by deranging the Nrf2/HO-1 signaling pathway.

**Conclusions:**

Ferrostatin-1 improved the rehabilitation of spinal cord injury mice by regulating ferroptosis through the Nrf2/HO-1 signaling pathway.

**Supplementary Information:**

The online version contains supplementary material available at 10.1186/s40001-023-01264-7.

## Background

Spinal cord injury (SCI) is a devastating neurological disorder with high mortality and disability rates [1]. For 30 years, its global prevalence has increased from 236 to 1298 cases per million in different countries [2, 3]. Despite its relatively small total numbers compared to cancer and coronary artery disease, this condition places a significant economic burden on individuals, families, and society. The annual costs associated with it are estimated at 7.7 billion dollars, while the lifetime costs for each patient exceed 3 million dollars [4, 5]. The psychological impact of adapting from a healthy individual to a paraplegic one can be devastating, especially since SCI typically affects young people. Those patients with SCI are two to five times more likely to die than those without SCI, especially in low-income countries. In clinical practice, SCI treatment usually includes methylprednisolone therapy, surgical decompression, and physical rehabilitation [6]. Despite numerous basic research studies on SCI, the complexity of pathophysiological changes makes it challenging to control the development of cascades, leading to poor treatment outcomes [7]. Therefore, knowledge of SCI pathophysiology and event sequences, including primary and secondary injury, is important for designing a suitable intervention for SCI.

It has been demonstrated that regulated cell death plays a crucial role in SCI, and the impact of different types of lethal cascades varies in their effect on SCI [8, 9]. Ferroptosis is a type of cell death characterized by iron requirement and reactive oxygen species (ROS) accumulation [10]. The process involves activating, expressing and regulating a series of genes. It was first studied in cancer and then found in many other diseases [11]. Recent studies discovered that ferroptosis plays a vital role in SCI, and a ferroptosis inhibitor can effectively protect neurons and promote motor function recovery [12, 13]. In another similar study, zinc activated the Nrf2 pathway to inhibit ferroptosis occurrence and positively affected nervous system recovery [14].

Ferrostatin-1 is the first ferroptosis inhibitor, which can bind with lipid ROS and protect cells against lipid peroxidation [12]. In this study, we mainly analyzed the function of ferrostatin-1 in SCI both in vitro and in vivo. Therefore, this new direction may provide a new therapeutic theory and approach for recovering neurological function after SCI.

## Materials and methods

### Mice and SCI model

The mice were fed in the Department of Experimental Animal Science, Nanchang University, 3–5 individuals per cage, 12/12 light and night cycle, and free access to water and food. The incomplete SCI model was established by a modified Allen’s method with a 10 g, 2.5 mm diameter iron bar from a height of 25 mm. Successful modeling showed lower limb convulsion and localized hematoma of the injured spinal cord in mice. All animal experiments in this study were approved by The first affiliated hospital of Nanchang University Ethics committee (Number: CDYFY-IACUC-202209QR027).

**Fig. 1:**
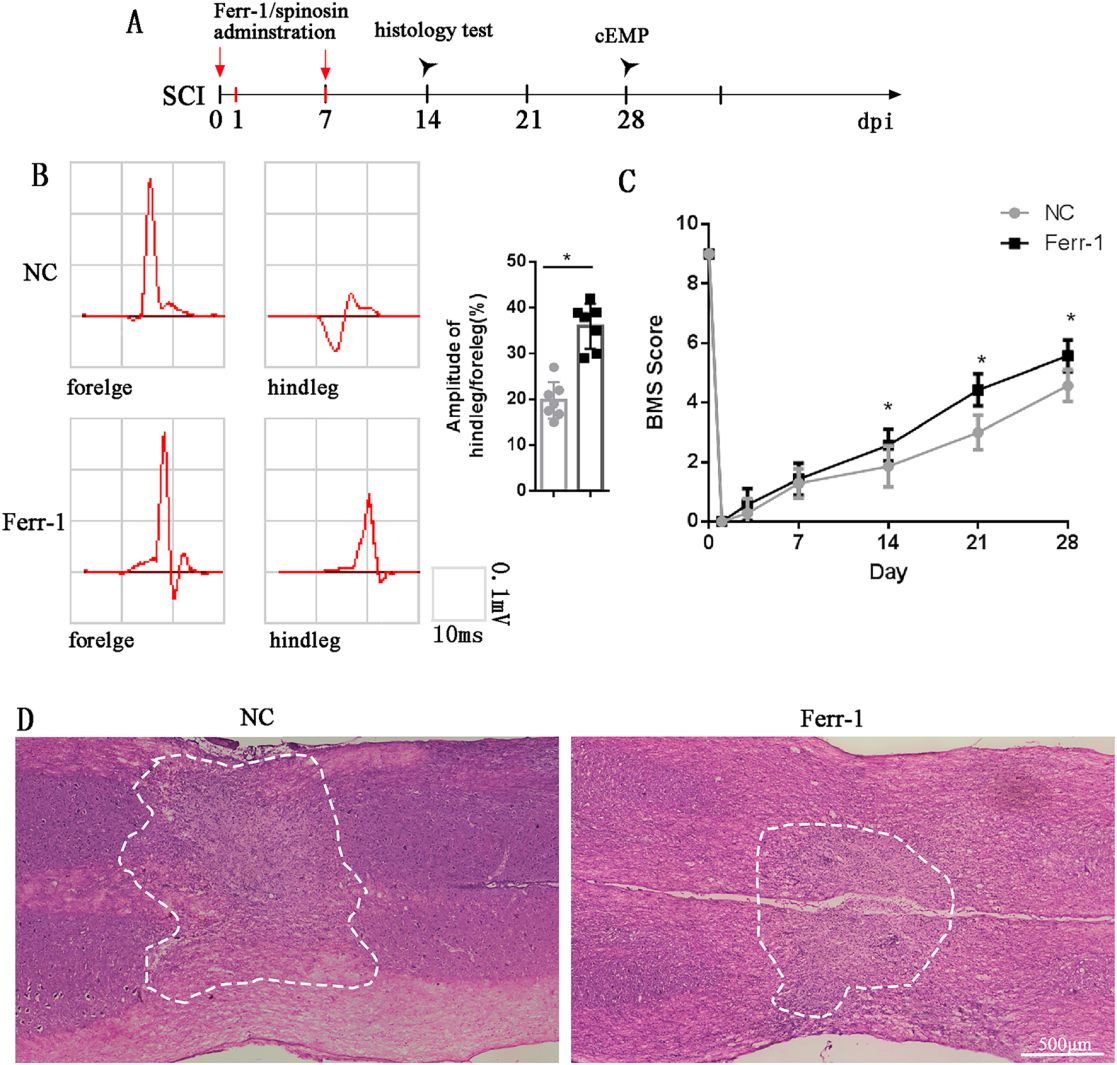
Ferrostatin-1 is conducive to rehabilitation of SCI. **A** Schematic diagram of experimental design, ferrostatin-1 or spinosin and ferrostatin-1 was administrated on the 1st and 7th day post-injury (dpi), BMS was indicated every 7 days, histology test including H&E, immunhistochemical and immunofluorescence were indicated on 14th dpi, cEMP was tested on 28th dpi. **B** Representative results of cEMP in the NC group and Ferrostatin-1 group. **C** The BMS results. **D** Representative H&E manifestation

All the drugs were administrated by intralumbar injection according to the reference [15]. The needle was inserted into the fourth and fifth lumbar intervertebral space, and the schematic diagram of the experimental design is shown in Fig. 1A.

### Histological and pathological assessments

The spinal cord tissue specimens were obtained 14 days after the SCI model was established, and frozen sections were prepared. Hematoxylin–eosin (H&E) staining was utilized to assess the histological manifestation. Immunofluorescence was applied to determine neuronal survival and scar formation after SCI. For the neuronal survival assessment, NeuN (ab177487, abcam) was used as the primary antibody, and Goat Anti-Rabbit IgG (H+L) Cross-Adsorbed Secondary antibody, Alexa Fluor 488 (A-11008, Thermo Fisher) was labeled as the secondary antibody. For the scar formation assessment, GFAP (ab7260, abcam) was used as the primary antibody, and Goat Anti-Rabbit IgG H&L (Alexa Fluor® 488) (ab150077, abcam) was labeled as the secondary antibody. The Nissl body was evaluated using the Nissl staining kit (G1434, Solarbio) to assess the state of the neurons.

### Western blot

The injured spinal cord was harvested and digested, and the tissue lysate protein concentration was determined using a bicinchoninic acid (BCA) protein quantitation kit (Thermo Scientific). After conventional electrophoretic separation, membrane transfer, and blocking, primary antibodies against Glutathione Peroxidase 4 (GPX4) (ab252833, abcam), HO-1 (ab305290, abcam), Nrf2 (ab92946, abcam) and secondary antibodies were sequentially applied. Pierce ECL Plus Western Blotting Substrate was indicated to assess the fluorescence intensity, and ImageJ software was used for quantitative identification.

### Iron concentration assessment

The tissue iron assay kit (ab83366, abcam) was indicated to detect the iron concentration in the spinal cord after injury. Local spinal cord tissue was obtained at each time point after SCI. After homogenization, the procedure was performed according to the product instructions. The absorbance was measured at 593 nm band, then the iron content in the tissues was calculated.

### Neurophysiological examination

Reference to the studies of Li et al., neuro electrophysiological examination was used to identify the neurological rehabilitation of mice. Briefly, the mice to be tested were anesthetized by intraperitoneal injection of 0.3% pentobarbital sodium. The cortical evoked motor potential (cEMP) was measured using the BL-420F Biological Function Experiment System. The mice were fixed in the prone position on the manipulator, and the stimulation electrodes were inserted subcutaneously at the corresponding locations. The main locations were as follows: the body projection of the cortical motor area: 1 mm from the right side of the fontanelle and 1 mm from the lower edge of the coronal line; the location of the recording electrode: the distal sciatic nerve of the left thigh to record the muscle action potential caused by electrical stimulation; the location of the reference electrode: the subcutaneous of the corresponding lower limb. The stimulation parameters detected by the bio-skill experimental system software cEMP were as follows: stimulation type: coarse voltage; stimulation mode: single stimulation; delay time: 100 ms; wave width: 1.0 ms; wave interval: 10 ms; waveform: square wave; frequency: 10 Hz; stimulation intensity: 3 V; gain: 20 times; scanning speed: 6.25 ms/div.

### Basso mouse scale (BMS)

The BMS system was used as an indicator to assess the neurological function of the lower extremities in mice after SCI. Mice were placed in softly lit, quiet rooms without external disturbances to assess the BMS score. The mice were placed on a horizontal table, and their lower limb movement joints and trunk were observed during free walking (a continuous walking distance of more than three times its body length was considered a valid score, and if there were interruptions and pauses during walking, the score need to be re-evaluated).

### Statistics analysis

All data were expressed as mean ± standard error of the mean (SEM), and two groups were compared using two-tailed Student’s *t*-test. GraphPad Prism 6 software (GraphPad Software, San Diego, CA) was utilized for data analysis and visualization.

## Results

### Ferrostatin-1 is conducive to the rehabilitation of SCI

To investigate the effects of ferrostatin-1 on neurological function in mice after SCI, we first assessed the mice’s neurophysiological performance of mice 28 days after SCI. The cEMP results demonstrated that the cEMP performance in Ferrostatin-1 group was significantly better compared to the NC group after SCI (Fig. 1A, B). Similarly, the BMS scores also exhibited a similar trend. At 14 days after SCI, the Ferrostatin-1 group had higher BMS scores in contrast with the NC group, which continued until our observation endpoint of 28 days (Fig. 1C). To further investigate the effect of ferrostatin-1 on the injured spinal cord, we analyzed spinal cord tissue obtained 14 days after SCI. H&E staining revealed that the Ferrostatin-1 group had a smaller injury area than the NC group (Fig. 1D).

### Ferrostatin-1 facilitates neuron survival and reduces scar formation after SCI

**Fig. 2:**
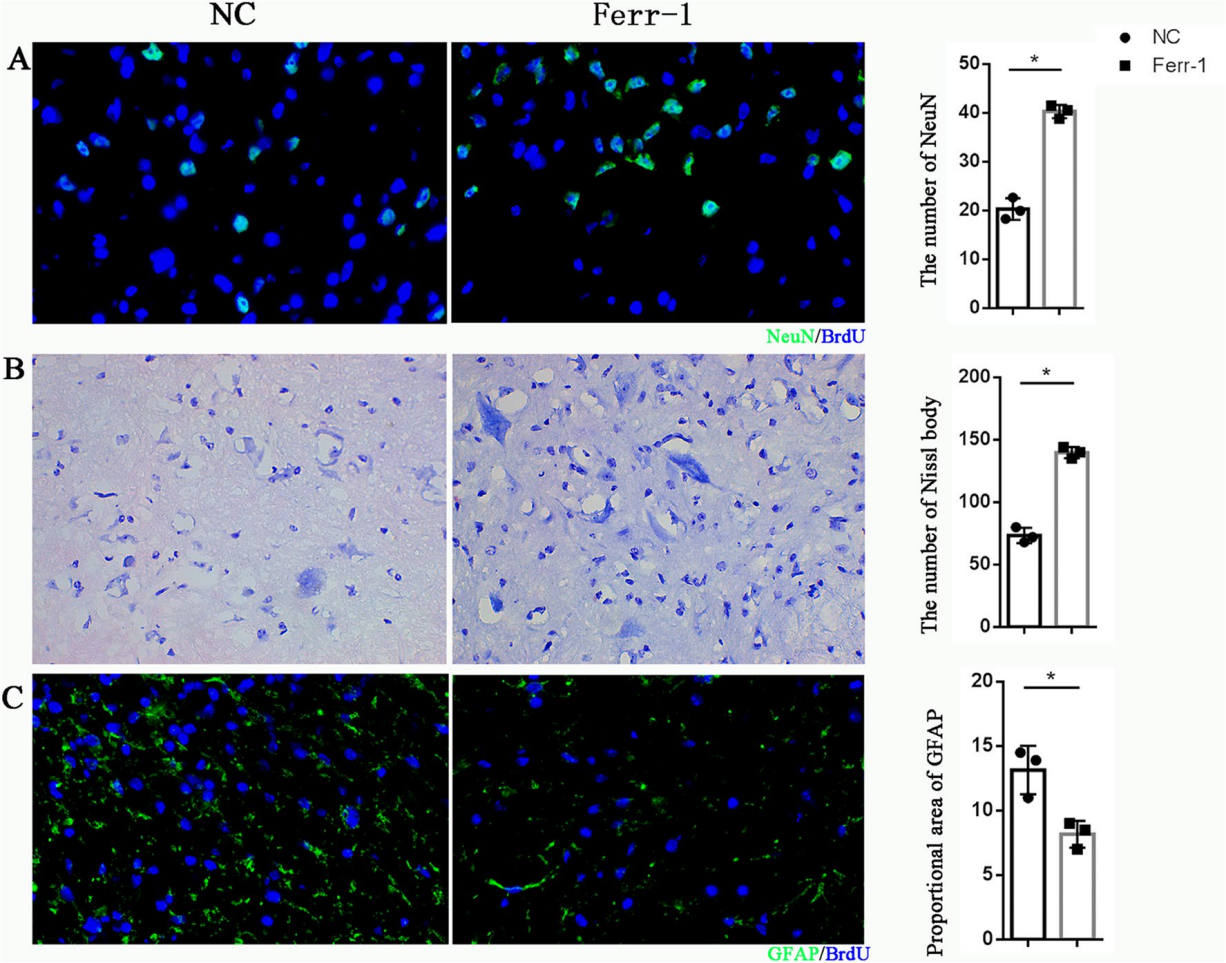
Ferrostatin-1 facilitates neuron survival and reduces scar formation after SCI. **A**–**C** Representative result of NeuN immunofluorescence manifestation (**A**), Nissl’s staining (**B**), and GFAP (**C**)

To further elucidate the pathological basis for promoting neurological function recovery in SCI mice by ferrostatin-1, we evaluated the histological changes in spinal cord tissue 14 days after SCI. By labeling the neuronal characteristic marker NeuN, we found that the Ferrostatin-1 group had more surviving neurons (Fig. 2A). Similarly, Nissl’s staining showed a similar trend (Fig. 2B). This further indicated that ferrostatin-1 was beneficial for neuronal survival after SCI. In addition, immunofluorescence staining of the glial cell marker GFAP revealed that ferrostatin-1 significantly inhibited glial cell proliferation after injury (Fig. 2C).

### Ferrostatin-1 regulates the Nrf2/HO-1 signaling pathway during ferroptosis

**Fig. 3:**
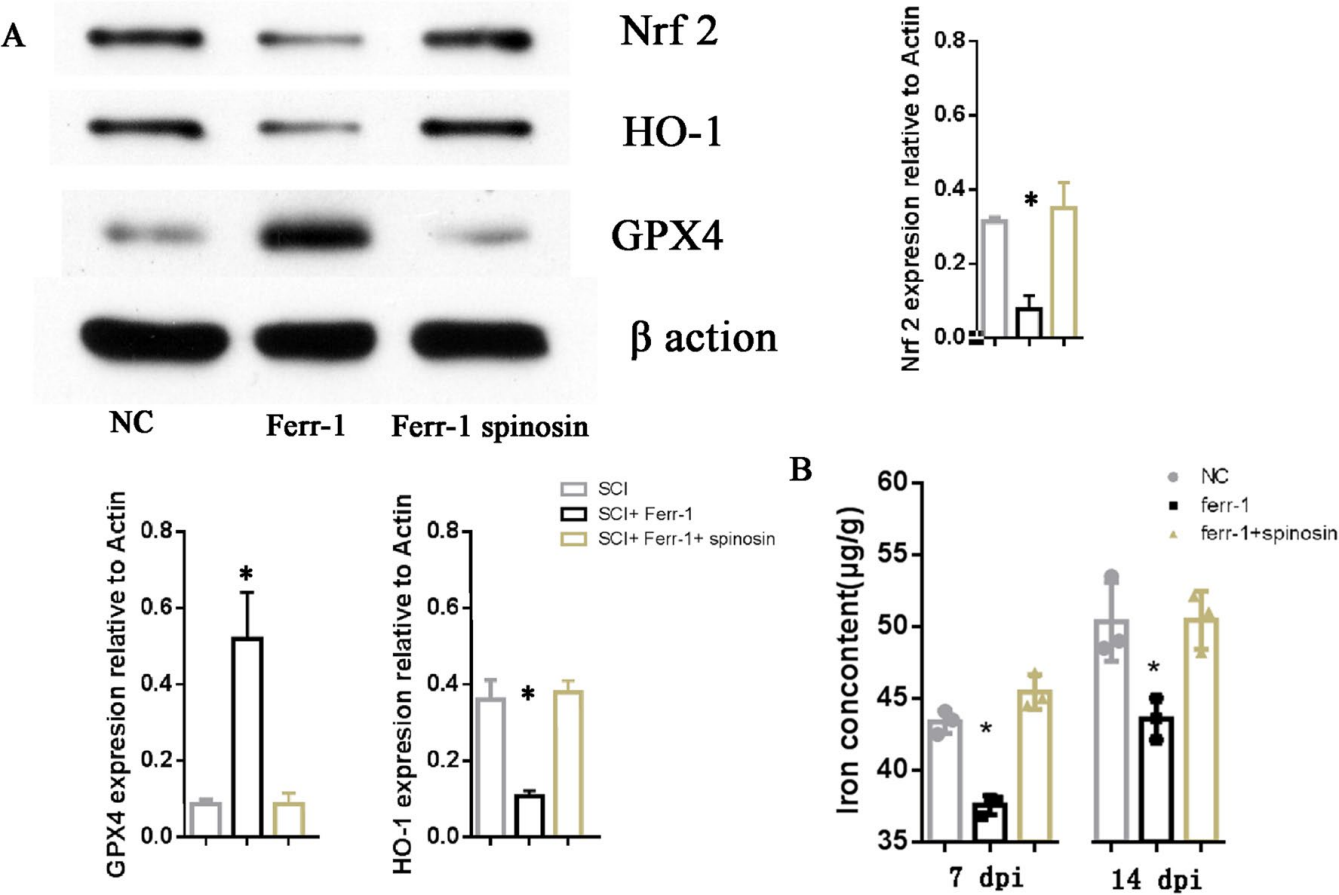
Ferrostatin-1 promotes GPX4 to disturbe Nrf2/HO-1 signaling pathway during ferroptosis. **A** Representative results of western blot of Nrf2/HO-1 signaling pathway change with Ferrostatin-1 and Ferrostatin-1 + Spinosin administration. **B** The quantification of Fe^2+^ concentration in injured spinal cord

Ferrostatin-1 is a selective inhibitor of ferroptosis. To confirm the role of ferrostatin-1 in SCI, we verified the relationship between ferrostatin-1 and the key Nrf2/HO-1 signaling pathway associated with ferroptosis, and introduced the selective small molecule activator Spinosin of the Nrf2/HO-1 pathway as a tool to restore ferroptosis after ferrostatin-1 blockade. The results showed that ferrostatin-1 significantly inhibited the activation of Nrf2/HO-1, and this inhibitory effect could be restored by Spinosin (Fig. 3A, additional file 1). In addition, we further measured the Fe^2+^ concentration in the injured tissue after SCI. The results showed that ferrostatin-1 significantly reduced the Fe^2+^ concentration on the 7th and 14th day post-injury, and Spinosin could also restore the local Fe^2+^ concentration after injury (Fig. 3B).

### Spinosin reverses the histological improvements of ferrostatin-1 after SCI

**Fig. 4:**
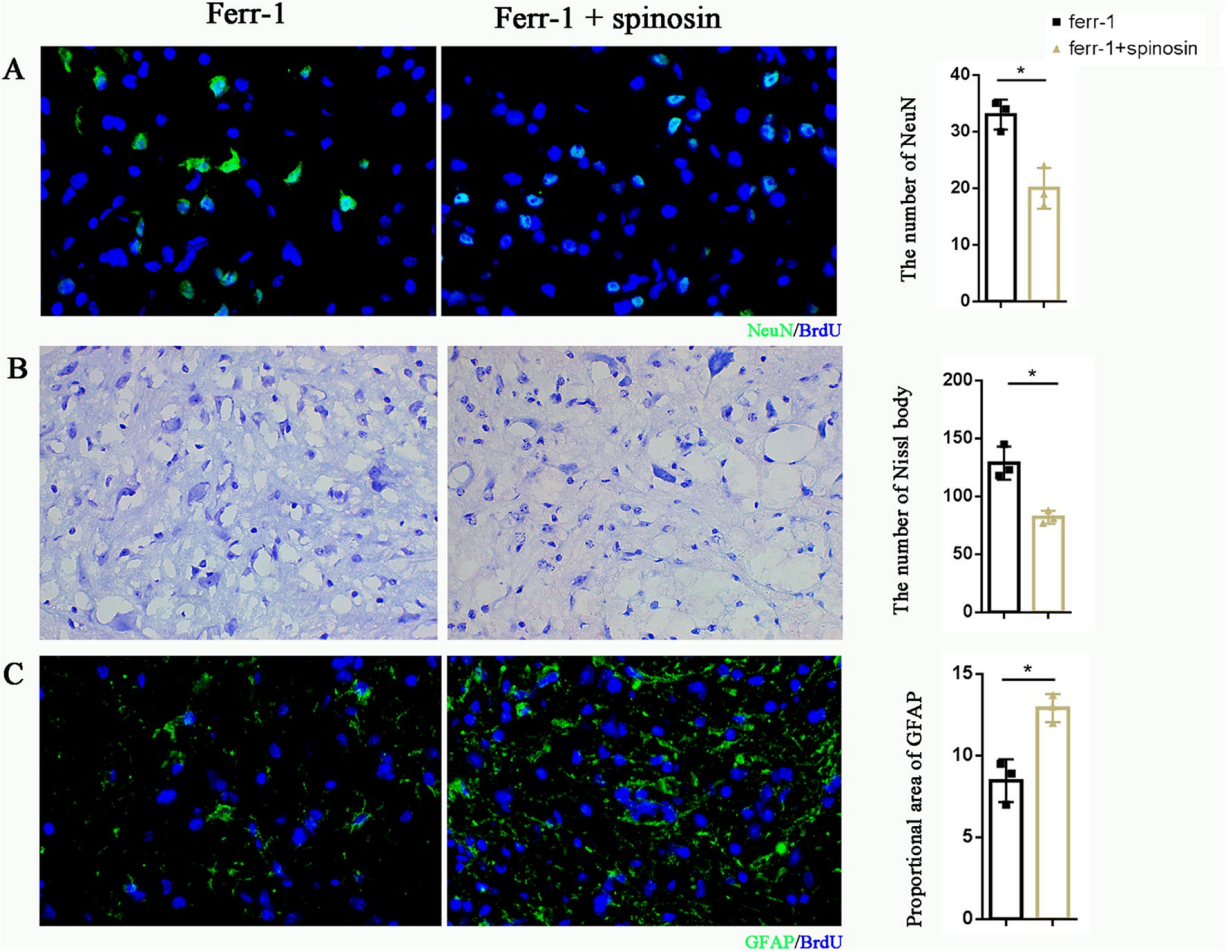
Spinosin abolishes the histological benefits of ferrostatin-1 administration at injured spinal cord. **A** Representative result of NeuN immunofluorescence manifestation (**A**), Nissl’s staining (**B**), and GFAP (**C**)

To further elucidate that ferrostatin-1 improved histological changes after SCI through the Nrf2/HO-1 pathway, Spinosin was administered simultaneously with ferrostatin-1. The results showed that NeuN and Nissl bodies were significantly lower in the Ferrostatin-1 + Spinosin group compared to the Ferrostatin-1 group. Additionally, GFAP expression was significantly higher in the Ferrostatin-1 + Spinosin group than in the Ferrostatin-1 group (Fig. 4). This suggested that after ferrostatin-1 inhibition of ferroptosis, restoration of the Nrf2/HO-1 pathway could block its effects on improving histological changes after SCI.

### Spinosin inhibits the neurological function improvement caused by ferrostatin-1

**Fig. 5:**
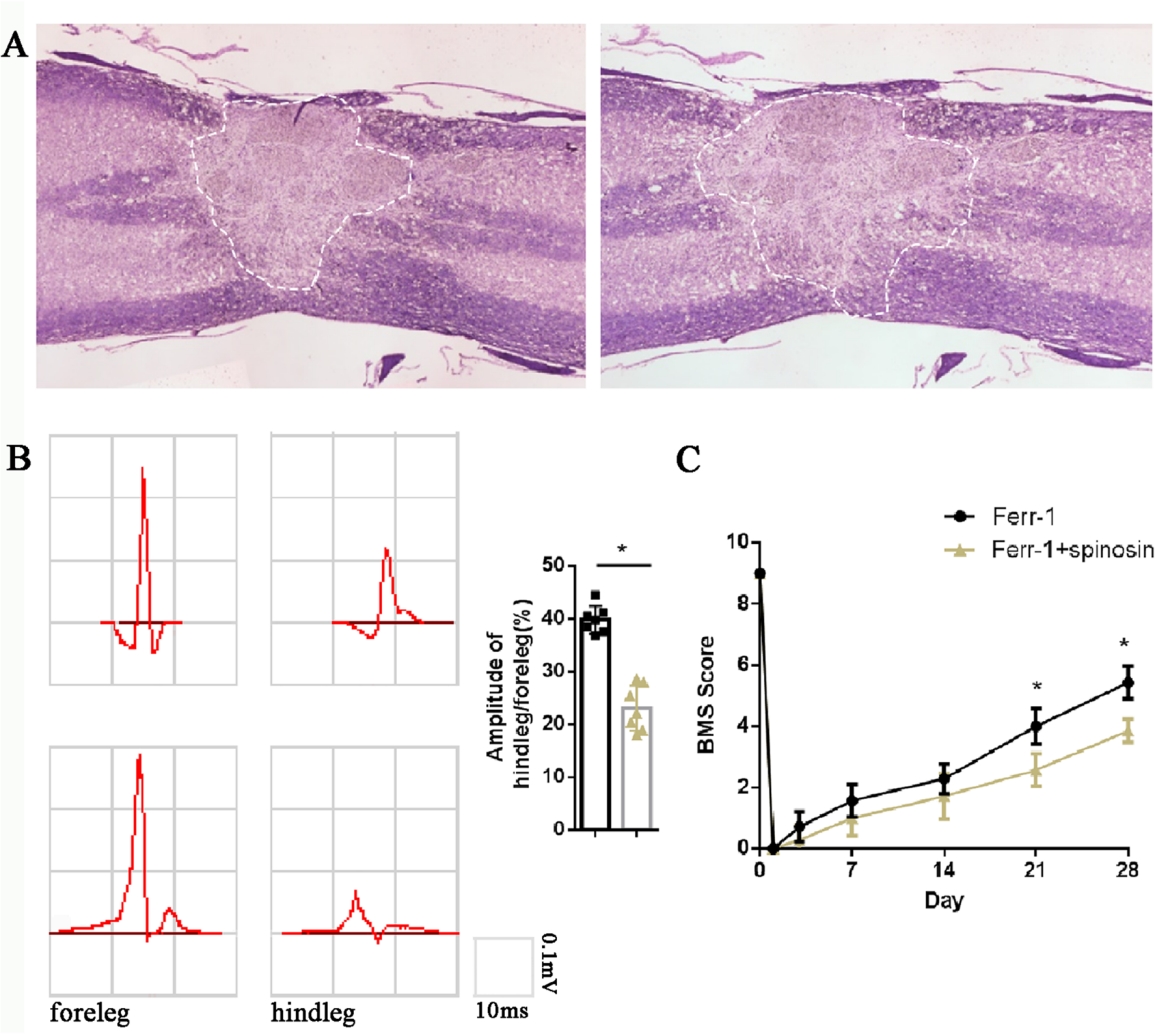
Spinosin neutralizes the therapeutic effects of ferrostatin-1 administration on neurological functional rehabilitation. **A** Representative H&E manifestation. **B** Representative result of cEMP in the Ferrostatin-1 group and Ferrostatin-1 + Spinosin group. **C** The BMS results

**Fig. 6:**
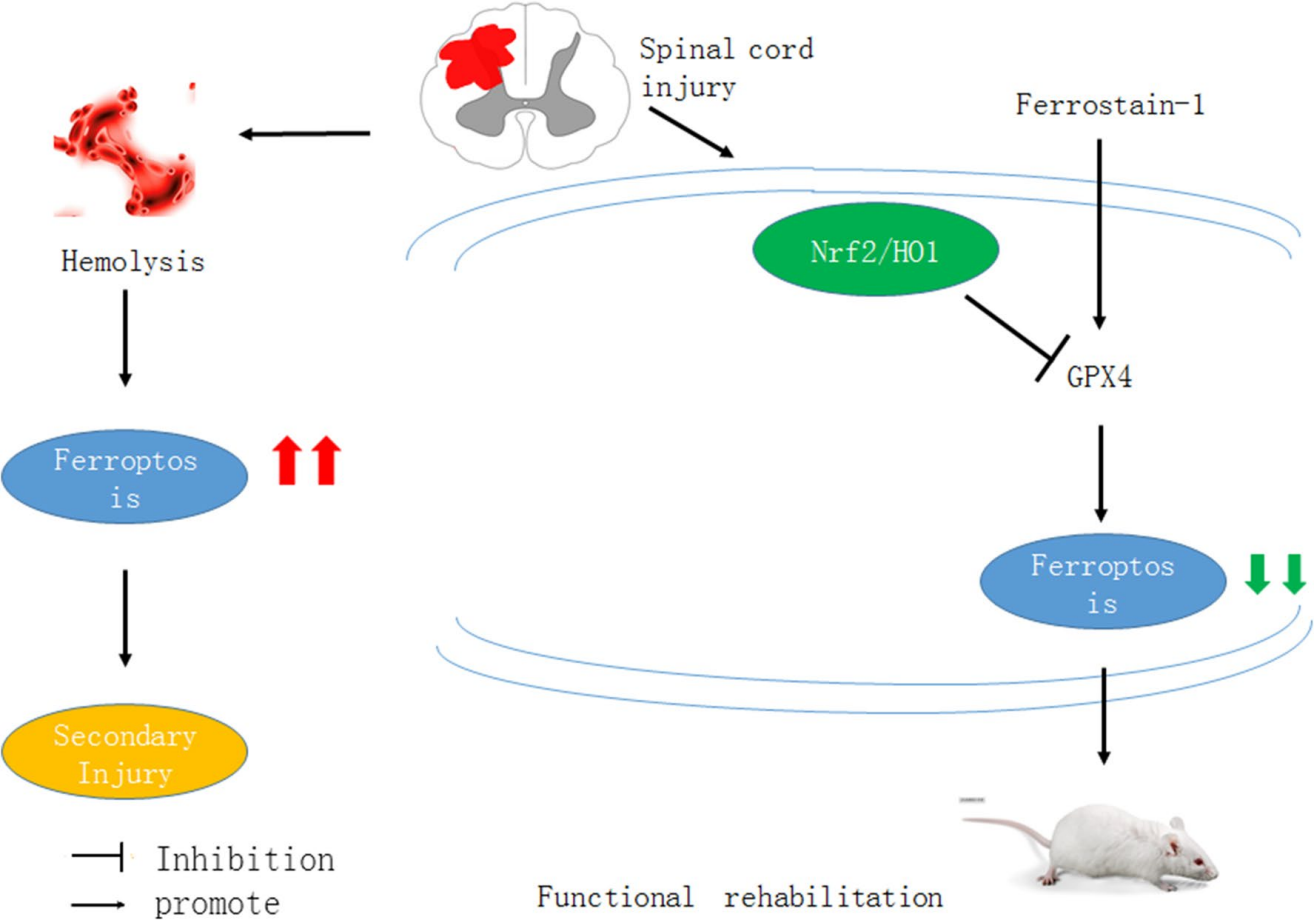
A schematic diagram of the experiment

To further demonstrate that ferrostatin-1 improved neurological function recovery after SCI by inhibiting ferroptosis, Spinosin was used again, and its effects on ferrostatin-1 administration were evaluated. The results showed that in the Ferrostatin-1 + Spinosin group, the injury area was larger, and both cEMP and BMS scores were significantly weaker in contrast with the Ferrostatin-1 group (Fig. 5). This section may be divided by subheadings. It should provide a concise and precise description of the experimental results, their interpretation, as well as the experimental conclusions that can be drawn (Fig. 6).

## Discussion

SCI is a major medical challenge facing humanity, and its injury process can be divided into primary and secondary injuries. The bottleneck of treatment lies in effectively dealing with the massive death of nerve cells caused by secondary injury [16]. Therefore, it is crucial to effectively control secondary injury after SCI, promote remaining neurons’ survival, protect neurological function, and promote SCI repair. Although the secondary injury mechanism is currently unclear, the generation of ROS and lipid peroxidation after injury are important reasons for secondary injury. Previous studies have shown that ferroptosis plays a vital role in SCI [17, 18]. In this study, the ferroptosis inhibitor, ferrostatin-1, contributed to the rehabilitation of SCI in vivo, with BMS scores in mice being higher than the NC group within 28 days’ post-injury, and the area of SCI being smaller than the NC group. In vitro experiments showed that ferrostatin-1 could reduce the local Fe^2+^ concentration after SCI and improve the neuronal status after SCI to ensure the survival of more neurons and inhibit scar formation. Further mechanism studies showed that ferrostatin-1 regulated ferroptosis in secondary injury after SCI by inhibiting the Nrf2/HO-1 signaling pathway. This is the first study of the role of the ferroptosis inhibitor ferrostatin-1 in SCI and provides new ideas for future SCI recovery.

Ferroptosis was first defined in 2012 as a regulated form of iron-catalyzed necrosis, which occurs through excessive peroxidation of polyunsaturated fatty acids and is distinct from previously discovered apoptosis, necrosis, and autophagy [10]. Initially, apoptosis and necrosis were considered the two primary types of cell death after SCI [3]. With the discovery of ferroptosis, more and more research has shown its important role in secondary damage after SCI. After SCI, primary injury causes vascular rupture, cell damage, and disruption of the blood–spinal cord barrier, which triggers a cascade of injury, including massive bleeding, release of inflammatory and apoptotic factors in the acute phase, red blood cell aggregation, cell fragmentation, and hemolysis, all of which can lead to Fe^2+^ overload at the site of injury [19, 20]. In addition, emergency injury causes a large amount of ROS, which increases the toxic excitability of glutamate and excessive lipid peroxidation. These factors interact to cause ferroptosis. In 2019, Yao et al. preliminary confirmed the existence of ferroptosis in the early stage of SCI through a rat model of SCI. They found that the iron content, HNE, GPX4, xCT protein levels, and mitochondrial morphology all changed in SCI. Intervention with iron chelator in SCI rats could inhibit the occurrence of ferroptosis, increase neuron survival, alleviate morphological changes in spinal cord tissue, and promote spinal cord function recovery [21]. Zhang et al. found through electron microscopy that ferroptosis-related manifestations appeared 15 min after injury. By using the iron-specific inhibitor SRS16-86 to intervene in SCI rats, they reduced lipid peroxide products, enhanced endogenous antioxidant capacity, and inhibited ferroptosis, thereby promoting spinal cord function recovery [12]. Our research was similar to previous results. Within 28 days after SCI, Fe^2+^ and MDA levels were significantly elevated. On the first day after modeling, GPX4 was significantly downregulated and 4HNE was significantly upregulated, indicating the occurrence of ferroptosis after SCI.

With the discovery of ferroptosis in SCI, more and more scholars have tried to inhibit this process from observing the recovery of spinal cord function. (−)-Epigallocatechin-3-gallate could reduce the death rate of cerebellar granule neurons by increasing the phosphorylation level of PKD1, while inhibiting ferroptosis to promote recovery after SCI [22]. Another study investigated the role of proanthocyanidin in which it was found that the proanthocyanidin-treated group significantly reduced the level of iron accumulation, while also reducing TBARS, ACSL4, and Alox15B and increasing the levels of GSH, GPX4, Nrf2, and HO-1 in the injured spinal cord, which led to the improvement of spinal cord function [23]. These reports demonstrated that iron inhibitors could inhibit ferroptosis from promoting recovery in SCI.

The core of ferroptosis is that damage to the GSH peroxidase system in cells leads to large amounts of lipid ROS accumulation in the cell, inducing cell death. GPX4 is the second largest GSH peroxidase in mammals and is an important regulatory upstream factor in ferroptosis. It can reduce lipid peroxides to their corresponding alcohol forms and interrupt the chain reaction of lipid peroxidation [24, 25]. Chen et al. induced motor neuron degeneration and paralysis in mice by conditionally knocking out the GPX4 gene in neurons, suggesting that ferroptosis could accelerate the progression of SCI [26]. Nrf2 is a key regulator of cellular antioxidant response and plays a key role in preventing lipid peroxidation and ferroptosis. Under normal conditions, Nrf2 in the cell nucleus induces the transcription of the HO-1 gene and upregulates HO-1 protein expression. There is evidence that ferroptosis-related diseases may result from abnormal Nrf2 signaling. A large number of experimental evidence suggests that the Nrf2/HO-1 pathway is involved in the occurrence and development of ferroptosis, and when the Nrf2/HO-1 signaling pathway is activated, ferroptosis in cells is inhibited, and damaged cells and abnormally metabolized lipids are improved [27, 28].

Ferrostatin-1 is a selective iron inhibitor that can remove iron-generated lipid ROS by inhibiting iron-catalyzed oxidation and enzyme-mediated iron-dependent catalysis. Through the intervention of ferrostatin-1, we found that it could significantly promote the recovery of nerve function after SCI. Firstly, the specimens obtained after modeling showed that the injury area of the SCI group formed a cavity, with a large number of cell infiltrations and scar formation. In contrast, the cavity, scar area, and neuron survival in the Ferrostatin-1 group were significantly smaller than those in the SCI group. Next, the BMS scores of mice in the Ferrostatin-1 group at different time points were higher than those in the pure SCI group. All of this indicated that the ferroptosis inhibitor plays a certain role in SCI. Further experimental results suggested that in GPX4, an important upstream regulator of ferroptosis, and key signaling pathway Nrf2/HO-1 of ferroptosis, through the introduction of ferrostatin-1 and the small molecule activator Spinosin, it was found that the inhibitory effect of ferrostatin-1 and the local Fe^2+^ concentration can be restored by small molecule activator, and histologically and behaviorally also have partial recovery effect.

There are some limitations in the present study. First, there is no direct evidence that ferrostatin-1 inhibits neuronal apoptosis after SCI in vitro, and second, there is no specific mechanism of inhibiting scar hyperplasia by ferrostatin-1. However, the article also provided a new direction in the treatment of SCI.

## Conclusions

In summary, this study provides in vivo evidence that the ferroptosis inhibitor ferrostatin-1 could improve the rehabilitation neurological functional rehabilitation after SCI, by interaction with the classical Nrf2/HO-1/GPX4 signaling pathway (Fig 6).

### Supplementary Information

**Additional file 1:** The original Western blot data of Nrf2/HO-1 signaling pathway change with Ferrostatin-1 and Ferrostatin-1 + Spinosin administration.

## Data Availability

Not applicable.
