# Supplementary figures and images for: Ferrostatin-1 facilitated neurological functional rehabilitation of spinal cord injury mice by inhibiting ferroptosis
Source: Eur J Med Res. 2023 Sep 11;28:336. doi: 10.1186/s40001-023-01264-7 (PMC10494332; doi:10.1186/s40001-023-01264-7)

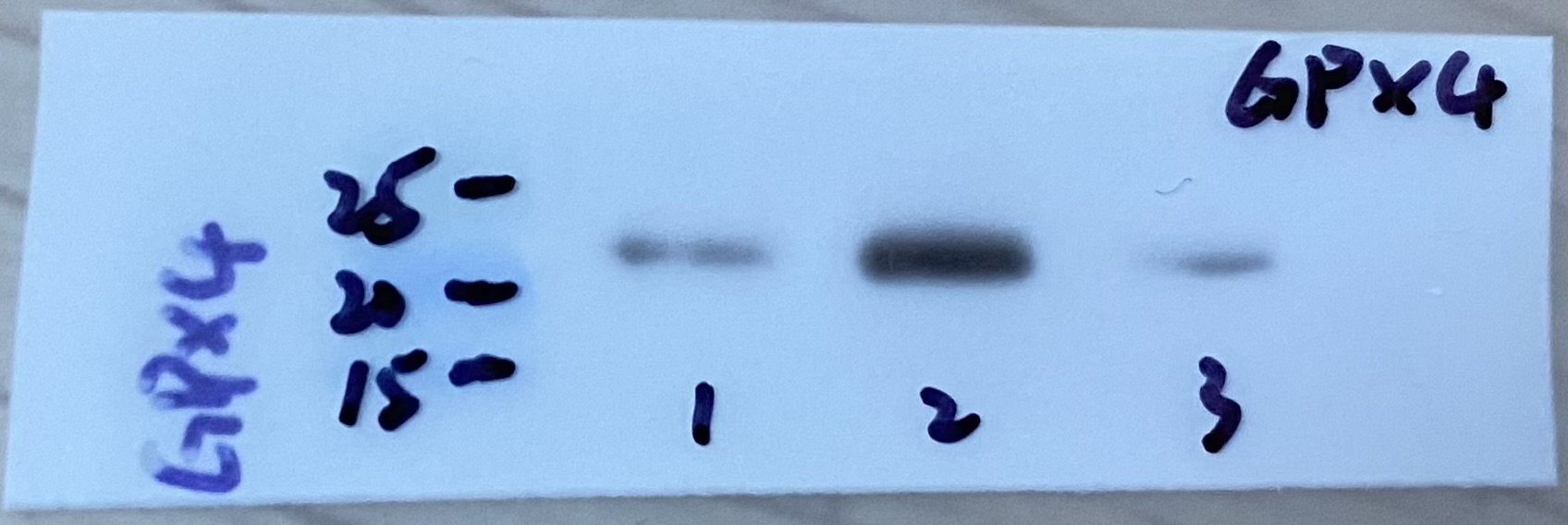

Supplement: Supplementary file 1 — Additional file 1: The original Western blot data of Nrf2/HO-1 signaling pathway change with Ferrostatin-1 and Ferrostatin-1 + Spinosin administration. [file 40001_2023_1264_MOESM1_ESM.zip › figure 3 WB/GPX4-1.JPG]

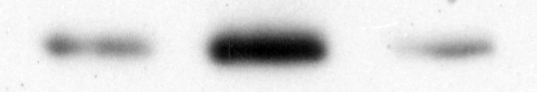

Supplement: Supplementary file 1 — Additional file 1: The original Western blot data of Nrf2/HO-1 signaling pathway change with Ferrostatin-1 and Ferrostatin-1 + Spinosin administration. [file 40001_2023_1264_MOESM1_ESM.zip › figure 3 WB/GPX4-1灰.tif]

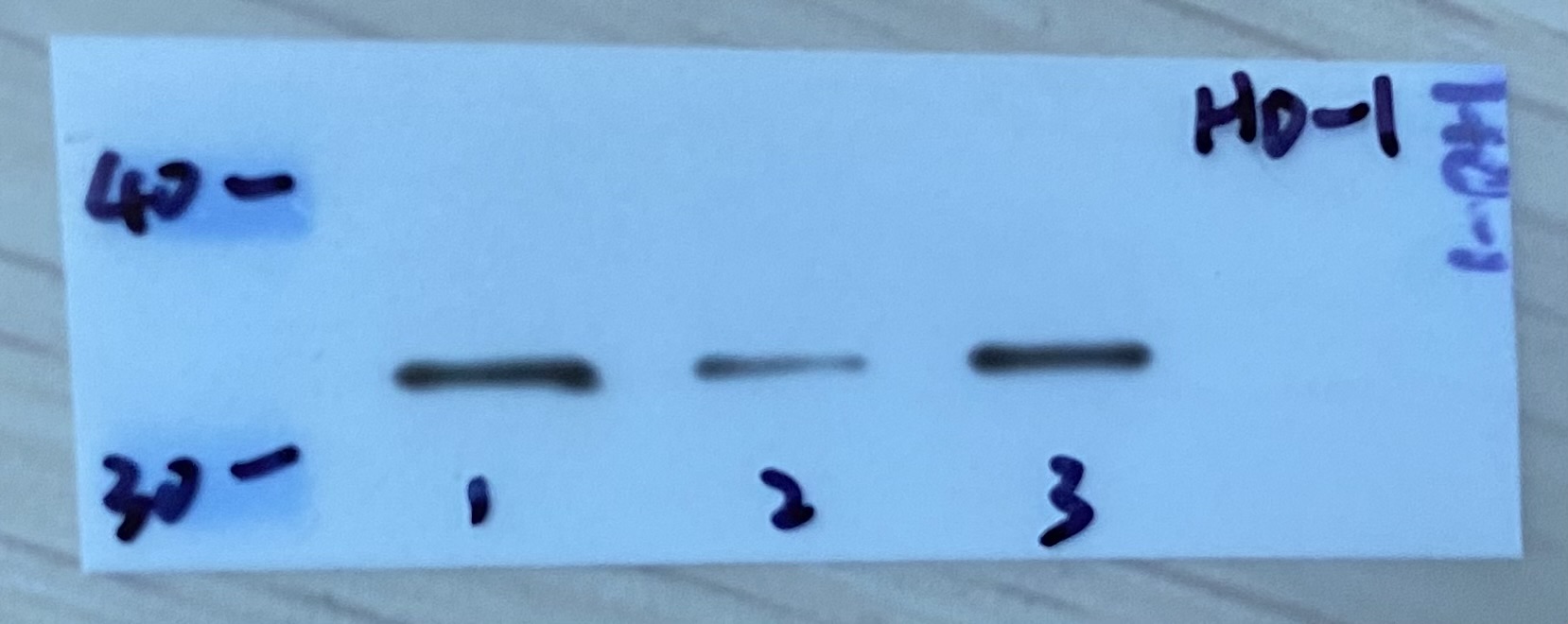

Supplement: Supplementary file 1 — Additional file 1: The original Western blot data of Nrf2/HO-1 signaling pathway change with Ferrostatin-1 and Ferrostatin-1 + Spinosin administration. [file 40001_2023_1264_MOESM1_ESM.zip › figure 3 WB/HO-1-1.JPG]

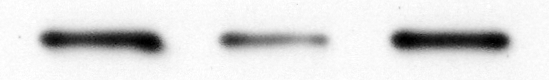

Supplement: Supplementary file 1 — Additional file 1: The original Western blot data of Nrf2/HO-1 signaling pathway change with Ferrostatin-1 and Ferrostatin-1 + Spinosin administration. [file 40001_2023_1264_MOESM1_ESM.zip › figure 3 WB/HO-1-1灰.tif]

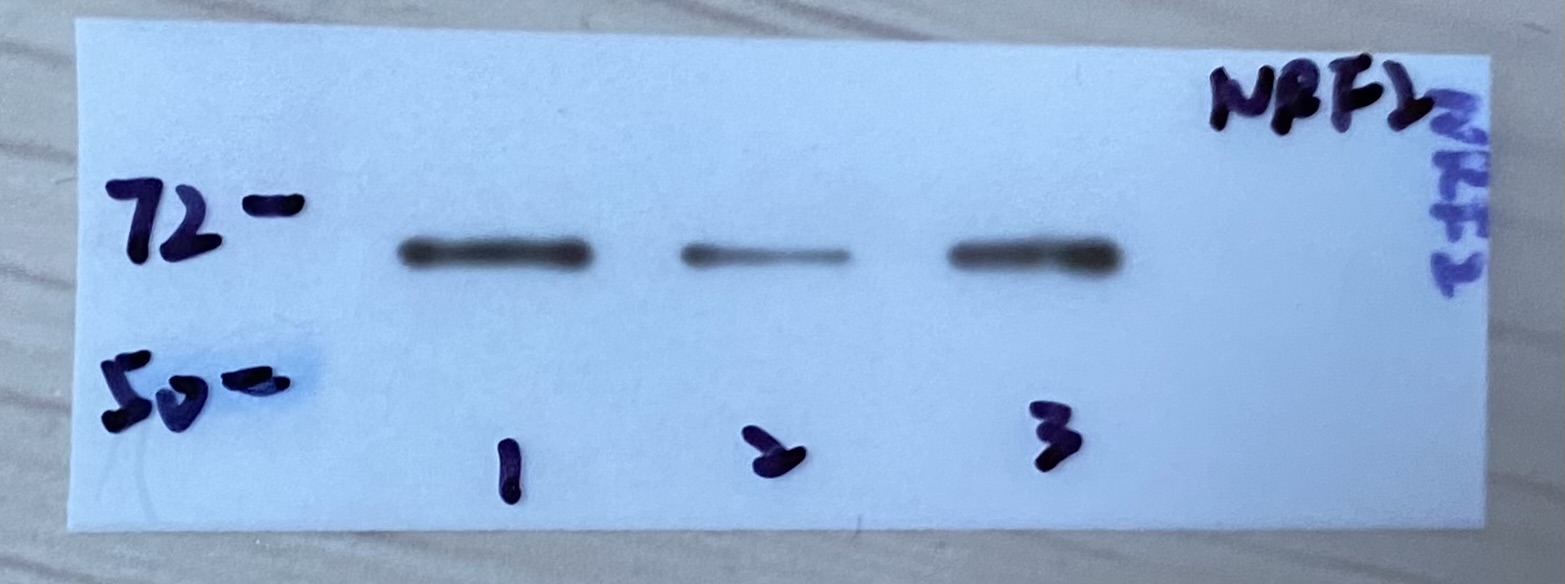

Supplement: Supplementary file 1 — Additional file 1: The original Western blot data of Nrf2/HO-1 signaling pathway change with Ferrostatin-1 and Ferrostatin-1 + Spinosin administration. [file 40001_2023_1264_MOESM1_ESM.zip › figure 3 WB/NRF2-1.JPG]

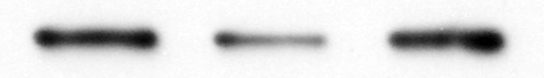

Supplement: Supplementary file 1 — Additional file 1: The original Western blot data of Nrf2/HO-1 signaling pathway change with Ferrostatin-1 and Ferrostatin-1 + Spinosin administration. [file 40001_2023_1264_MOESM1_ESM.zip › figure 3 WB/NRF2-1灰.tif]

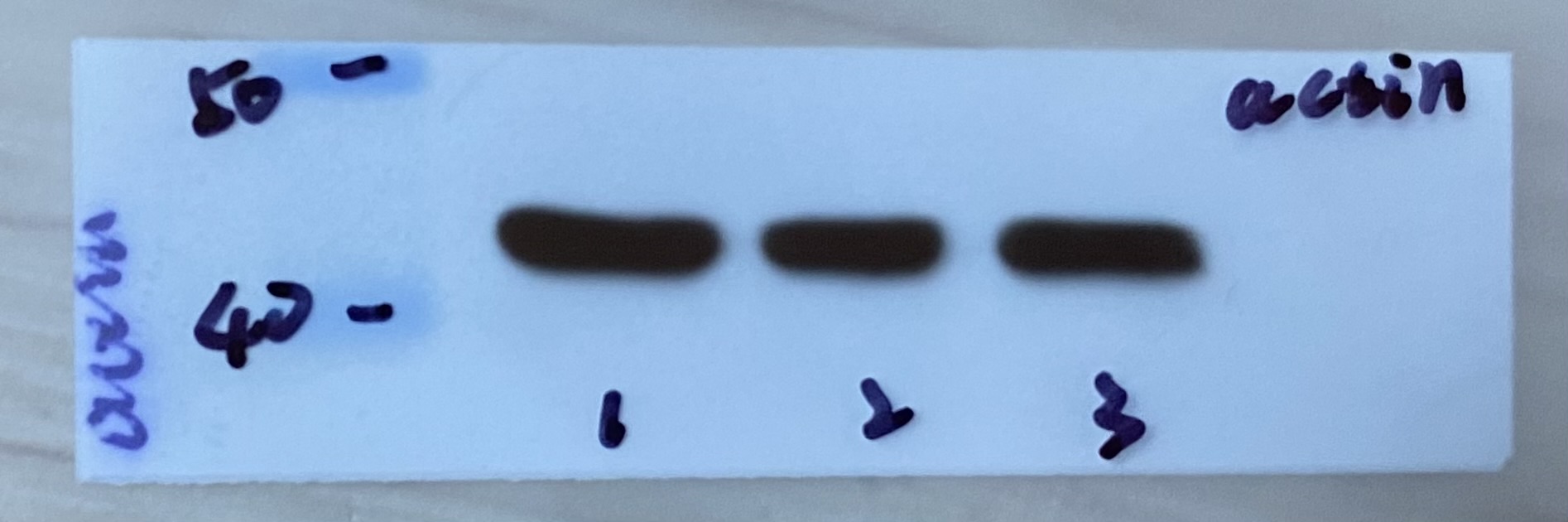

Supplement: Supplementary file 1 — Additional file 1: The original Western blot data of Nrf2/HO-1 signaling pathway change with Ferrostatin-1 and Ferrostatin-1 + Spinosin administration. [file 40001_2023_1264_MOESM1_ESM.zip › figure 3 WB/actin-1.JPG]

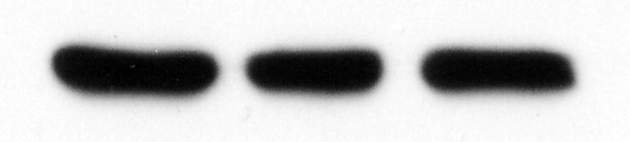

Supplement: Supplementary file 1 — Additional file 1: The original Western blot data of Nrf2/HO-1 signaling pathway change with Ferrostatin-1 and Ferrostatin-1 + Spinosin administration. [file 40001_2023_1264_MOESM1_ESM.zip › figure 3 WB/actin-1灰.tif]
